# Wrist-Measured Nighttime Home BP and Left Ventricular Hypertrophy: The WISDOM-HMOD Study

**DOI:** 10.1161/HYPERTENSIONAHA.125.26510

**Published:** 2026-05-11

**Authors:** Kazuomi Kario, Kenji Harada, Yusuke Ishiyama, Takeshi Fujiwara, Keisuke Narita, Yusuke Suzuki, Hiroyuki Mizuno, Ryosuke Komi, Naoko Tomitani, Satoshi Hoshide

**Affiliations:** Division of Cardiovascular Medicine, Department of Medicine, Jichi Medical University School of Medicine, Tochigi, Japan (K.K., K.H., Y.I., T.F., K.N., Y.S., H.M., N.T., S.H.).; Komi Internal Clinic, Tokyo, Japan (R.K.).

**Keywords:** blood pressure, heart failure, humans, risk factors

## Abstract

**BACKGROUND:**

Nighttime blood pressure (BP) assessed by ambulatory BP monitoring is linked to left ventricular hypertrophy and its sequelae, including heart failure. However, the association between nighttime BP measured by a wrist-type oscillometric home BP monitor―designed to be less intrusive than brachial devices―and left ventricular hypertrophy remains unclear.

**METHODS:**

The WISDOM-HMOD study (Wrist ICT-based Sleep and Circadian Blood Pressure Monitoring Program-Hypertension–Mediated Organ Damage), a substudy of the WISDOM-Night study, included 1218 patients with hypertension or heart failure (mean age, 67.2±12.0 years; 53.9% men) who were recruited between 2021 and 2024. Nighttime BPs (2:00, 3:00, and 4:00 am and 4 hours after bedtime) and morning/evening BPs over 7 days were measured using a wrist-type home BP monitor (HEM9601T; Omron).

**RESULTS:**

The average nighttime systolic/diastolic BP was 110.5±13.0/63.5±8.8 mm Hg, and 70.4% of patients had well-controlled nighttime BP (<120/70 mm Hg). The average left ventricular mass index assessed by echocardiography was 90.1±23.6 g/m^2^ in men and 80.3±19.3 g/m^2^ in women. An increase in nighttime systolic BP was significantly associated with a higher left ventricular mass index, independent of office BP, morning home BP, and evening home BP. Furthermore, elevated nighttime systolic BP was a risk factor for left ventricular hypertrophy in both sexes (odds ratio per 10-mm Hg increase, 1.36 [95% CI, 1.15–1.60] overall; 1.54 [95% CI, 1.19–2.00] in men; and 1.27 [95% CI, 1.01–1.58] in women), independently of covariates including office and morning home BP.

**CONCLUSIONS:**

This study shows that nighttime home BP measured using a less sleep-disturbing wrist-type device is an independent risk factor for left ventricular hypertrophy and its potential sequelae, including new-onset or recurrent heart failure, with high-risk nighttime BP thresholds differing by sex.

**Novelty and Relevance**

**What Is New?**

Higher nighttime home blood pressure (BP) measured with a wrist-type oscillometric BP monitor is an independent risk factor for left ventricular hypertrophy (LVH), distinct from morning BP.
The threshold of wrist-measured nighttime BP associated with an increased risk of LVH differed between men and women; however, elevated wrist-measured nighttime SBP was a significant determinant of LVH in both sexes.
Among patients receiving ≥3 antihypertensive medications, uncontrolled home BP was significantly associated with LVH, whereas a similar association was not observed in those receiving 2 or fewer medications. No significant association was observed between office BP and LVH in any group.

**What Is Relevant?**

Nighttime BP is an established prognostic marker in hypertension management, but its assessment has been limited by the burden of ambulatory blood pressure monitoring. This study provides evidence that nighttime BP can be reliably assessed using a wrist-type home BP monitoring device under conditions resembling routine clinical practice.
Identification of sex-specific nighttime BP thresholds and the prognostic significance of uncontrolled wrist-measured nighttime BP may inform individualized risk stratification and treatment strategies in patients with hypertension.
The absence of an association between office BP and LVH underscores the clinical importance of home-based nighttime BP monitoring.

**Clinical/Pathophysiological Implications?**

Wrist-measured nocturnal BP obtained with a device that causes less discomfort and sleep disturbance may be a sensitive tool for identifying patients at high risk of LVH and its sequelae, such as heart failure.
Implementing nighttime home BP monitoring into routine hypertension management could provide a more comprehensive approach to cardiovascular risk assessment.

Nighttime blood pressure (BP) is a better predictor of target organ damage and cardiovascular events than daytime BP.^1,2^ While ambulatory BP monitoring (ABPM) remains the gold standard for evaluating nighttime BP profiles, home BP monitoring (HBPM) devices with automated nocturnal BP measurement functions have recently emerged, and evidence supporting their use has been accumulating. HBPM devices for nighttime BP evaluation offer several advantages over ABPM, such as the ability to perform repeated monitoring, less restriction (no need to wear the device during daytime activities), and less sleep disturbance (typically fewer measurements per night). However, even with HBPM devices for nighttime BP evaluation, the need to wear an upper-arm cuff during sleep and the pressure applied to the upper arm during measurement can still cause discomfort and sleep disturbance, potentially leading to overestimation of true BP during sleep.^3,4^ A recently developed wrist-cuff oscillometric BP monitor (HEM-9601T; Omron Healthcare, Kyoto, Japan) measures BP with less measurement noise and less cuff compression compared with a conventional upper arm-type oscillometric device. The accuracy of BP measurements obtained with this device in the supine position has been confirmed under both laboratory conditions^5^ and home-sleep conditions.^6^ In a comparative study of nighttime BP measurement using this wrist device and an upper-arm HBPM device, the wrist device caused less frequent nocturnal awakening than the upper-arm device.^6^

Recent studies using nocturnal HBPM have primarily adopted either fixed-time measurements (eg, 2:00–4:00 am) or measurements based on elapsed time after bedtime, both of which have demonstrated clinical relevance for cardiovascular risk prediction.^7,8^ On the basis of this evidence, we used a measurement schedule that integrates both fixed-time and bedtime-based approaches in the present study.

Left ventricular hypertrophy (LVH) is the most potent predictor of adverse cardiovascular outcomes in the hypertensive population, and is an independent risk factor for cardiovascular disease, including heart failure.^9^ Nighttime BP measured by ABPM has been reported to be associated with LVH and subsequent heart failure.^10^ However, whether nighttime BP measured by a less intrusive wrist-type oscillometric device is associated with LVH remains unknown. We hypothesized that nighttime BP measured by this wrist-type device is associated with LVH. Confirmation of this hypothesis would indicate that nighttime BP measurement with the wrist-type device is useful for identifying individuals with LVH, similarly to ABPM-based nighttime BP measurement. Accordingly, this study aimed to investigate the relationship between LVH assessed by echocardiography and wrist-measured BP.

## Methods

### Data Availability

The data sets analyzed during the current study are part of an ongoing research project. Therefore, they are not publicly available at this stage. However, data may be available from the corresponding author upon reasonable request after study completion.

### Study Design

The WISDOM-HMOD study (Wrist ICT-based Sleep and Circadian BP Monitoring Program-Hypertension–Mediated Organ Damage) is a substudy of the WISDOM-Night study (WISDOM-Night BP; Figure S1). Details of both study designs have been published previously^11^ and are provided in the Supplemental Material. The study protocol was approved by the Jichi Medical University Hospital Bioethics Committee for Clinical Research. The study was registered on a clinical trials registration site (University Hospital Medical Information Network Clinical Trials Registry no. UMIN000043599). The WISDOM-Night study, which includes the WISDOM-HMOD study, is an ongoing multicenter, prospective study that enrolled patients between 2021 and 2024. The present report analyzes baseline data from the WISDOM-HMOD study.

### Study Participants

Participants of the WISDOM-HMOD study were recruited from WISDOM-HMOD study-participating centers (Jichi Medical University Hospital, Washiya Hospital, and Komi Internal Clinic). Patients with hypertension (treated patients or untreated patients with office BP ≥130/80 mm Hg) or heart failure were eligible for this study. Individuals were excluded if they engaged in shift work, were using a pacemaker, had a recent history of cardiovascular events (within the past 6 months), had chronic renal failure requiring hemodialysis, or had chronic renal failure or other serious diseases (eg, cancer, collagen disease). In addition, participants with congenital heart disease, as well as those with suspected hypertrophic cardiomyopathy or valvular disease, were excluded from the analysis of this study.

### BP Measurements

HBPM was performed using a validated wrist-cuff oscillometric HBPM device (HEM-9601T; Omron Healthcare) designed for both nighttime use and the conventional daytime use of HBPM. Details of the device are described in the Supplemental Material. Participants were instructed to measure their BP at home for 7 days, including 2 consecutive seated self-measurements each morning and before bedtime, and 1 preset automatic nocturnal BP measurement at each of 4 scheduled time points—2:00, 3:00, and 4:00 am and 4 hours after bedtime (thereby combining fixed-time and bedtime-based measurements). Morning and evening BP were self-measured in a sitting position with the wrist device positioned at heart level. Participants were asked to measure morning BP within 1 hour after waking, following urination, before taking any medications, and before eating breakfast. Evening BP was measured before going to bed. The device was set to automatically record BP measurements at the 4 preset time points (2:00, 3:00, and 4:00 am and 4 hours after bedtime). Unless otherwise specified, nighttime BP refers to the average of 4 readings throughout this article.

Seated office BP was measured twice at each clinic visit, at the upper arm position, using a validated oscillometric device, with a 1-minute interval between measurements.

### Echocardiography

Participants in the WISDOM-HMOD study underwent echocardiography at baseline and at the 1- and 3-year follow-up visits performed by trained doctors or technicians at each participating institute (Figure S1). All centers followed prespecified standardized imaging and measurement protocols. DICOM files and raw loops were digitally archived. Readers were blinded to clinical information. Site initiation/training and archiving of key loops were required.

Left ventricular mass (g) was calculated using the following formula^12^: 0.8 (1.04× [(LVIDD + PWTD + IVSTD)^3^ – (LVIDD)3])+0.6, where LVIDD is the left ventricular internal diameter at end-diastole, PWTD is the posterior wall thickness at end-diastole, and IVSTD is the interventricular septal thickness at end-diastole. Left ventricular mass was indexed to body surface area (Du Bois formula, m^2^), and LVH was defined as a left ventricular mass index (LVMI) >115 g/m^2^ in men and >95 g/m^2^ in women.^13^ For sensitivity analyses, a uniform LVMI cutoff value of 100 g/m^2^ applied to both sexes was also used.^14^

To ensure consistency of echocardiographic measurements across participating centers, a comprehensive quality control program was implemented. Details of the quality control and reproducibility of echocardiographic measurements are provided in the Supplemental Methods. Interobserver, intraobserver, and interinstitution reproducibility were evaluated using a randomly selected subset of echocardiographic data sets obtained from this multicenter study (n=30). Measurement reproducibility was assessed using intraclass correlation coefficients, calculated with a 2-way random-effects model with absolute agreement (correlation coefficient [2,1]), and coefficients of variation. Acceptable reproducibility was predefined as a correlation coefficient >0.85 for left ventricular ejection fraction and volumes and a coefficients of variation <10% for key echocardiographic parameters, in accordance with previously published quality standards for multicenter echocardiographic studies.^15,16^

### Statistical Analysis

Data are presented as the mean±SD or SE for continuous variables and as percentages for categorical variables. For comparisons between 2 groups, we used the *t* test for continuous variables and the χ^2^, Fisher exact test, or McNemar test for categorical variables. The McNemar test was used to compare paired categorical classifications obtained from different nighttime BP averaging indices in the same participants. To test the trend among groups stratified by the number of antihypertensive medications, we used a *t* test for the slope in linear regression models or the Cochran-Armitage test. In addition, Bonferroni correction was applied for multiple comparisons. To examine the linear association of LVMI with BP parameters, Pearson correlation coefficients were used. Differences between correlations were tested using the Fisher *Z* test. Multivariable linear regression models and multivariable logistic regression models were used to analyze the associations of BP indices with LVMI and LVH, respectively.

In all analyses, a 2-sided *P* value of <0.05 was considered statistically significant. All statistical analyses were performed using SAS software (version 9.4; SAS Institute, Inc).

## Results

Among the 2710 participants in the WISDOM-Night study, 1392 patients also agreed to participate in the WISDOM-HMOD study. After excluding participants with congenital heart disease, as well as those with suspected hypertrophic cardiomyopathy or valvular disease, data from a total of 1218 participants were ultimately used for the analysis in the WISDOM-HMOD study (Figure S1).

The characteristics, BP levels, and left ventricular assessment of the 1218 patients, as well as those stratified by sex, are presented in Table 1. Overall, the mean age was 67.2±12.0 years, with no significant difference between sexes. All patients had hypertension. Male patients had higher BMI and a higher prevalence of current smoking, current drinking, diabetes, atrial fibrillation, hyperuricemia, sleep apnea syndrome, heart failure, and history of cardiovascular disease than female patients, and all of these differences were statistically significant. Morning systolic BP (SBP)/diastolic BP and nighttime SBP/diastolic BP were significantly higher in males. The prevalence of LVH was significantly higher in females. Nighttime BP averages calculated from all 4 readings (2:00, 3:00, and 4:00 am and 4 hours after bedtime) and from the 3 fixed-time readings (2:00, 3:00, and 4:00 am) were compared (Figure S2). Although a statistically significant difference was found, 98.6% of patients had an absolute difference of ≤5 mm Hg between the 2 averages. The prevalence of nocturnal hypertension, defined as an average nighttime SBP/dystolic BP of 120/70 mm Hg or higher based on the 4-reading average, was 29.6%.

**Table 1.**
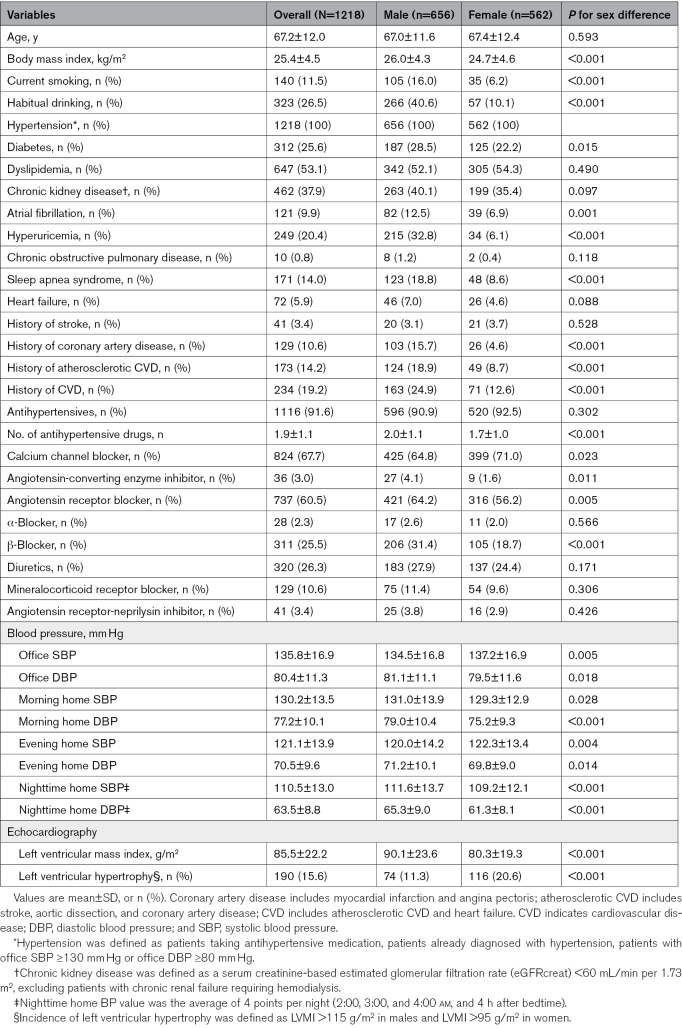
Baseline Characteristics, Blood Pressure, and Left Ventricular Parameters

The associations of LVMI with morning SBP (0.249, *P*<0.001) and nighttime SBP parameters (0.213–0.252, all *P*<0.001) were stronger than those with office SBP and evening SBP (Table 2). The associations between LVMI and diastolic BP parameters were weak (Table S1). Multivariable linear regression analysis showed that higher nighttime SBP was significantly associated with higher LVMI (2.44 g/m^2^ increase per 10-mm Hg increase in nighttime SBP, *P*<0.001), independent of other BP measures such as morning SBP and office SBP in the overall participants (Table 3). In sex-specific analyses, both nighttime and morning SBP were significantly associated with LVMI in both females and males. Similarly, elevated nighttime SBP was a risk factor for LVH in both sexes (odds ratio per 10-mm Hg increase, 1.36 [95% CI, 1.15–1.60] for all; 1.54 [95% CI, 1.19–2.00] for men; and 1.27 [95% CI, 1.01–1.58] for women), independently of covariates including office BP and morning home BP (Table 4). Consistent with the findings for LVMI, both nighttime and morning SBP were significantly associated with LVH in women, whereas in men only nighttime SBP remained significant. The Figure shows the association between nighttime BP levels and LVMI and LVH by sex. Compared with the lowest nighttime BP group (nighttime SBP <100 mm Hg), female participants with nighttime SBP ≥100 mm Hg and male participants with nighttime SBP ≥120 mm Hg had significantly higher LVMI (Figure [A], unadjusted model in Table S2). Similarly, the incidence of LVH was significantly higher in female participants with nighttime SBP ≥100 mm Hg and male participants with nighttime SBP ≥120 mm Hg (Figure [B]). When a uniform cutoff value of 100 g/m^2^ was applied to both sexes, the results were similar to those obtained when sex-specific definitions of LVH were used (Table S3).

**Table 2.**
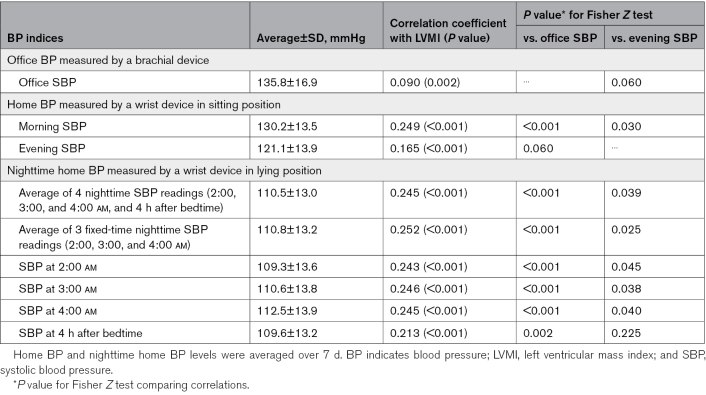
Associations Between Systolic Blood Pressure parameters and Left Ventricular Mass Index

**Table 3.**
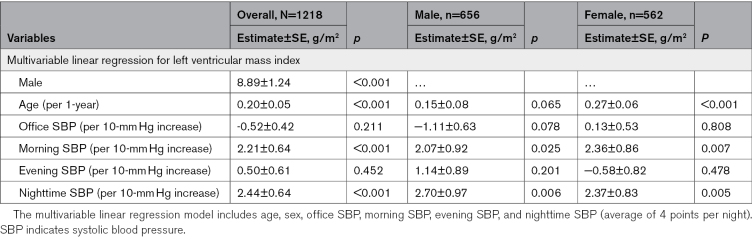
Multivariable Regression Analysis of Left Ventricular Mass Index

**Table 4.**
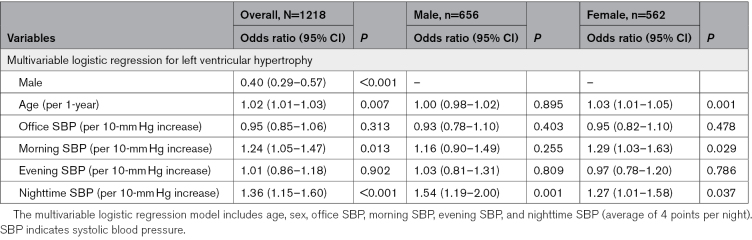
Multivariable Regression Analysis of Left Ventricular Hypertrophy

**Figure.**
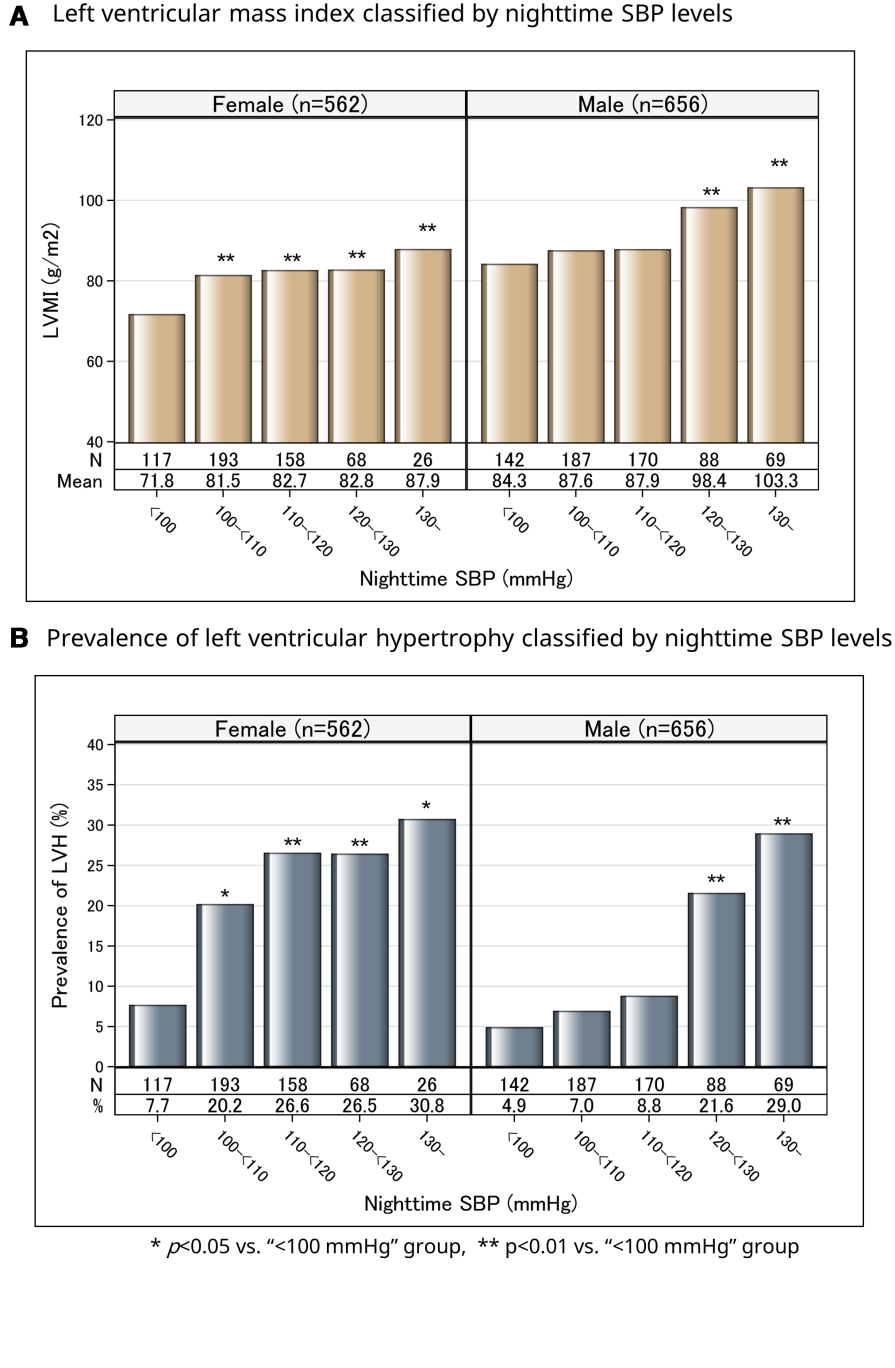
**Association between nighttime blood pressure (BP) levels and left ventricular structure by sex. A**, Left ventricular mass index (LVMI) classified by nighttime systolic blood pressure (SBP) levels. **B**, Prevalence of left ventricular hypertrophy (LVH) classified by nighttime SBP levels.

The association between nighttime SBP levels and LVMI remained consistent across all 3 models (the unadjusted model, the model adjusted for age and office SBP, and the model further adjusted for morning SBP) in the male participants. In the female participants with nighttime SBP ≥120 mm Hg, however, the association was no longer significant in the model that included age, office SBP, and morning SBP (Table S2). When stratified by morning SBP levels, the cutoff point for a significant increase in LVH prevalence was 135 mm Hg in females and 145 mm Hg in males (Table S4). Among participants with well-controlled morning SBP (<135 mm Hg), the associations between nighttime BP levels and LVH within each sex were similar to those in the overall participants (Table S5).

Table 5 shows the association between BP control status and the prevalence of LVH, stratified by the number of antihypertensive medications. In the overall participants, the prevalence of LVH increased with the number of antihypertensive medications. Among patients with uncontrolled home SBP (morning, evening, or nighttime), LVH prevalence was particularly high in patients receiving ≥3 antihypertensive medications. Although the proportion of patients with uncontrolled nighttime BP decreased with the number of antihypertensive medications, the prevalence of LVH increased linearly (Table S6).

**Table 5.**
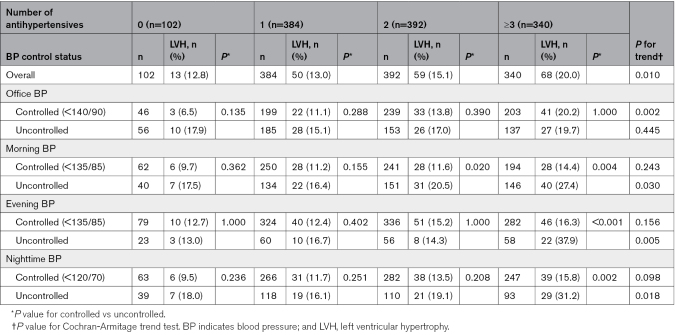
Comparison of the Prevalence of Left Ventricular Hypertrophy Between Blood Pressure Control Status Stratified by the Number of Antihypertensive Medications

Reproducibility of echocardiographic measurements is shown in Table S7.

## Discussion

This study is the first to show that nighttime home BP measured with a wrist-type oscillometric BP monitor that causes less sleep disturbance is an independent determinant of LVH, beyond morning BP. Although the nighttime BP threshold associated with an increased risk of LVH differed between men and women, higher nighttime SBP was a significant determinant of LVH in both sexes. Moreover, even among participants with well-controlled nighttime SBP (<120 mm Hg; 79.4% of the cohort), nighttime SBP remained significantly associated with LVH. In addition, among patients receiving ≥3 antihypertensive medications, uncontrolled BP was strongly associated with LVH.

### Association Between Wrist-Measured Home BP and LVH

Nighttime and morning home SBP, measured using a wrist-type oscillometric device, showed a significant positive correlation with LVMI. The results of the multivariable analysis, which included nighttime and morning home SBP together, showed that both were independent risk factors for an increased LVMI and the incidence of LVH. Even among participants with well-controlled morning SBP, higher nighttime SBP was associated with a higher prevalence of LVH, suggesting that nighttime BP monitoring may help identify residual cardiovascular risk that cannot be detected by morning BP alone, thereby supporting its clinical utility in the assessment of target organ damage.

A meta-analysis of echocardiographic studies using upper arm-type ABPM devices demonstrated that LVMI was significantly correlated with nighttime SBP but not with daytime SBP.^17^ In addition, a study of a general population showed that nighttime SBP measured by an upper arm-type ABPM device was an independent predictor of new-onset LV hypertrophy.^18^ Taking into account both the previous and current findings, nighttime BP, regardless of whether it is measured by an upper arm-type or wrist-type device, serves as a crucial index for predicting alterations in cardiac structure.

### Sex Differences in the Nighttime BP Threshold Associated With LVH Risk

Wrist-measured nighttime home SBP was significantly associated with the incidence of LVH in both sexes. However, the threshold BP levels associated with an increased risk of LVH differed between men and women. In men, the BP thresholds associated with an increased risk of LVH were 120 mm Hg for nighttime SBP and 145 mm Hg for morning SBP. In contrast, in women, the corresponding thresholds were lower: 100 mm Hg for nighttime SBP and 135 mm Hg for morning SBP.

Previous studies have reported sex differences in the relationship between left ventricular mass and nighttime BP and day-night dipping.^19,20^ An interventional study showed that hypertension-induced LVH in women was more resistant to antihypertensive therapy, and that regression of hypertrophy was more difficult to achieve in women than in men, even with well-controlled BP.^21^ Sex-related differences in LVH might be related to sex hormones.^22,23^ In the Framingham Study, the age-related increase in LVM in women was more pronounced in the postmenopausal period.^24^ Given that the mean age of our study participants was 67 years and that it likely included a substantial proportion of postmenopausal women, the observed sex differences in the association between LVH and BP may reflect the influence of age and hormonal status. These findings suggest that both sex and age should be carefully considered when evaluating the relationship between nighttime BP and LV structure.

### Pathophysiological Mechanisms Linking Nighttime BP Elevation to Organ Damage and Cardiovascular Disease

In the transition from hypertension to heart failure, hypertensive heart disease is widely recognized as 1 phenotype of cardiac dysfunction.^25^ In a Japanese nationwide ABPM study, the JAMP (Japan Ambulatory Blood Pressure Monitoring Prospective) study, elevated nighttime BP and abnormalities in BP circadian rhythm, such as a riser pattern, were associated with development of heart failure.^26^ Moreover, Cuspidi et al^18^ reported that nighttime BP measured by ABPM was associated with development of LVH in the PAMELA (Pressioni Arteriose Monitorate e Loro Associazioni) study.

Nighttime BP in the supine position is a more important contributor to cardiac overload than daytime BP in the upright position, because the supine position already increases left ventricular preload via increased venous return from the lower body.^10^ In fact, nighttime BP has been reported to be a stronger risk factor for heart failure than daytime BP.^26^ Moreover, increased sympathetic nervous activity has been observed in patients with a nondipper BP or a riser BP pattern, both of which are associated with elevated nighttime BP.^27^ Pathological conditions characterized by heightened sympathetic nervous activity, such as obesity, sleep apnea, diabetes, chronic kidney disease, or prevalent cardiovascular disease, are themselves independent risk factors for cardiovascular events.^28,29^ Therefore, nighttime BP elevation may exert a particularly large impact on cardiovascular risk in individuals with these underlying disorders.

### Impact of Antihypertensive Medication Use on LVH

For the participants receiving 0 to 2 antihypertensive medications, the prevalence of LVH was similar between those with well-controlled and uncontrolled home BP. However, among hypertensive patients taking ≥3 medications, the prevalence of LVH was significantly higher in patients with uncontrolled BP. In contrast, when patients were stratified according to office BP control status, no difference in LVH prevalence was observed even among those receiving ≥3 medications. These findings suggest that control of home BP—particularly morning and nighttime BP—is associated with LVH, whereas office BP alone may be insufficient for risk assessment. Among patients taking ≥3 antihypertensive medications, assessment of nighttime BP control is strongly recommended to identify those at high risk of treatment-resistant nocturnal hypertension.

### Limitations

Some limitations of the present study should be acknowledged. First, our study participants had relatively well-controlled nighttime SBP (79.4% of participants had nighttime SBP <120 mm Hg), which may have led to an underestimation of the association between nighttime SBP and LVMI or LVH. Therefore, our findings should be validated in populations with a higher prevalence of uncontrolled BP, in populations with comorbidities associated with elevated nighttime BP, such as diabetes or sleep apnea, and in more diverse racial and ethnic groups. Second, ethnicity-specific reference values for LVMI remain an important consideration. The applicability of uniform LVMI thresholds across different populations may be limited due to potential ethnic differences in cardiac structure.^30^ In the absence of universally established LVMI cutoff values specific to Japanese populations, we additionally performed sensitivity analyses using a sex-neutral threshold to assess the robustness of our findings. Third, individuals engaged in shift work or those who did not sleep between 2:00 am and 4:00 am were excluded from this study; however, many people have diverse sleep patterns. Our results should thus be validated in cohorts with heterogeneous sleep patterns. Finally, the cross-sectional nature of this baseline analysis precludes establishing a causal relationship between wrist-measured nighttime BP and HMOD.

### Conclusions

This is the first study to show that nighttime home BP measured with a wrist-type oscillometric BP monitor is independently associated with LVH and provides complementary information to morning BP. These findings suggest a potential role of nighttime BP in assessing cardiovascular risk in patients with hypertension.

### Perspectives

The present findings suggest that nighttime home BP monitoring with a wrist-type device has the potential to enhance risk stratification in patients susceptible to LVH and heart failure. Future studies with larger and more diverse populations are warranted to validate these results and to establish optimal threshold values for clinical application. Because wrist-type home BP monitors cause minimal restriction and sleep disturbance, nighttime BP measured by these devices may serve as a sensitive marker for identifying patients at high risk of heart failure or its recurrence. Although current hypertension management for out-of-office BP primarily relies on morning and evening home BP values, nighttime home BP has not yet been established as a major therapeutic target. In the future, integrating nighttime home BP control assessed by user-friendly home devices into routine clinical practice may represent a new direction for optimizing hypertension management.

## Article Information

### Acknowledgments

The authors thank all nurses and physicians at the participating centers and the study staff for their time, effort, and many contributions to this work.

### Disclosures

K. Kario has received research grants from Omron Healthcare Co., Ltd., A&D Co., Ltd., and Fukuda Denshi Co., Ltd. This study was coordinated by the Super Circulation Monitoring with High Technology R & D Center, which is a joint research center between Jichi Medical University School of Medicine and Omron Healthcare Co., Ltd, but the employees of Omron Healthcare were not involved in the analysis of results. The other authors report no conflicts.

## Supplementary Material


