# Supplementary material for: Wrist-Measured Nighttime Home BP and Left Ventricular Hypertrophy: The WISDOM-HMOD Study
Source: Hypertension. 2026 May 11;83(7):e26510. doi: 10.1161/HYPERTENSIONAHA.125.26510 (PMC13275078; doi:10.1161/HYPERTENSIONAHA.125.26510)

**SUPPLEMENTAL MATERIAL**

**Wrist-measured nighttime home BP and left ventricular hypertrophy: the WISDOM-HMOD Study**

Kazuomi Kario, MD, PhD ^1 *^, Kenji Harada, MD, PhD ^1^, Yusuke Ishiyama, MD, PhD ^1^, Takeshi Fujiwara MD, PhD ^1^, Keisuke Narita MD, PhD ^1^, Yusuke Suzuki, MD ^1^, Hiroyuki Mizuno, MD, PhD ^1^, Ryosuke Komi, MD, PhD ^2^, Naoko Tomitani, BSc, PhD ^1^, Satoshi Hoshide, MD, PhD ^1^

^1^Division of Cardiovascular Medicine, Department of Medicine, Jichi Medical University School of Medicine, Tochigi, Japan

^2^Komi Internal Clinic, Tokyo, Japan

***Corresponding author**: Dr. Kazuomi Kario, Division of Cardiovascular Medicine, Department of Medicine, Jichi Medical University School of Medicine, 3311-1 Yakushiji, Shimotsuke, Tochigi 329-0498, Japan.

Tel.: +81-285-58-7344; Fax: +81-285-44-2132; Email: [kkario@jichi.ac.jp](mailto:kkario@jichi.ac.jp)

## **WISDOM–Night STUDY GROUP**

**Principal Investigator**:

Kazuomi Kario (Jichi Medical University)

**Study Office**:

Satoshi Hoshide (Study Manager), Ryoko Nozue (Registry Management), Yuri Matsumoto (Data Management), Naoko Tomitani (Statistical Analysis).

**Investigators network** (42 investigators [23 institutions]):

**Hokkaido**: Naoki Nakagawa, Asahikawa Medical University Hospital; Hajime Kihara, Kihara Cardiovascular Internal Medicine Clinic; Hidekazu Shimizu, Kikonai Municipal Hospital; Fumihiko Takahashi, Rumoi City Hospital. **Tochigi**: Kazuomi Kario, Satoshi Hoshide, Masahisa Shimpo, Yasushi Imai, Kenta Okada, Hiroshi Funayama, Kenji Harada, Tomoyuki Kabutoya, Yukiyo Ogata, Takahiro Komori, Tomonori Watanabe, Kenichi Katsurada, Masashi Kamioka, Hayato Shimizu, Yusuke Oba, Ayako Yokota, Hiroaki Watanabe, Kana Kubota, Yusuke Ishiyama, Takeshi Fujiwara, Yusuke Suzuki and Keisuke Narita, Jichi Medical University School of Medicine*; Kazuomi Kario, Satoshi Hoshide, Kenichi Katsurada and Keisuke Narita, Washiya Memorial Hospital*; Takeshi Fujiwara, Tochigi Medical Center Shimotsuga. **Ibaraki**: Hiroyuki Mizuno, Yukiyo Ogata and Kenta Fujimura, Ibaraki Western Medical Center. **Tokyo**: Azusa Hashimoto, Akabane Central General Hospital; Ryosuke Komi, Komi Internal Clinic*; Shinji Koba, Showa University Dental Hospital Internal Medicine Clinic. **Kanagawa**: Hiromitsu Sekizuka, Yokohama Respiratory Clinic; Jisho Kojima, Kojima Clinic. **Nagano**: Toshinobu Saito, Japanese Red Cross Iiyama Hospital. **Gifu**: Hiroyuki Ohbayashi, Tohno Chuo Clinic. **Aichi**: Koki Haimoto, Haimoto Clinic. **Hyogo**: Yasuhisa Abe, Abe Internal Medicine Clinic. **Hiroshima**: Yoshiro Kato, Kotsubaki Clinic. **Yamaguchi**: Yoshio Matsui, Matsui Internal Clinic. **Kagawa**: Yumiko Fujita, Fujita Neurosurgical Clinic. **Fukuoka**: Shin Ueda, Ueda Internal Clinic; Keisuke Shinohara, Kyushu University Hospital.

**＊WISDOM–HMOD Study participating centers (3 institutions)**

# **SUPPLEMENTAL METHODS**

## **Study design**

The WISDOM–Night Study (Wrist ICT-based Sleep and Circadian Blood Pressure Monitoring Program–Night blood pressure Study) was designed (1) to investigate the prognostic impact of wrist-measured nighttime home blood pressure (BP) parameters obtained by a validated wrist-type oscillometric home BP monitoring (HBPM) device with a timer function for nocturnal measurement (HEM-9601T; Omron Healthcare, Kyoto, Japan); and (2) to explore the indexes of pathological nighttime BP with clinical relevance. Participants were recruited by general practitioners in Japan if they fulfilled the inclusion and exclusion criteria. Patients who had at least one cardiovascular risk factor or a history of cardiovascular disease and were >20 years of age were included. In addition, participants were required to have a wrist circumference of 13.5–21.5 cm. Patients were excluded if they met any of the following criteria: being a shift worker (engaged in night shifts ≥3 days per week); having a pacemaker; having a history of cerebrovascular disease (excluding asymptomatic or transient cases) within 6 months before consent; having chronic renal failure requiring hemodialysis; or having another serious illness (e.g., end-stage cancer or active connective tissue disease). At baseline, seated office BP monitoring at the upper arm was taken twice using a validated oscillometric device. Then, participants were provided with a validated wrist-type oscillometric nocturnal HBPM device and instructed in its use to measure their BPs at home. Home BP measurements using the wrist nocturnal HBPM device were conducted for 7 days, including two consecutive seated self- measurements each morning and before bedtime, and one preset automatic nocturnal BP measurement at each of four scheduled time points—2:00, 3:00, and 4:00 AM and 4 h after bedtime. Questionnaires on sleep and salt intake were completed at baseline.

The present analysis is based on baseline data; patients in this cohort are followed up annually for 7 years. At the one-year follow-up visit (and additionally at the third-year visit for participants in the WISDOM-HMOD study), participants are asked to measure their home BP following the same HBPM protocol and to complete the sleep and salt intake questionnaires. At each annual follow-up visit, data are collected on their office BP, medications, and the occurrence of CVD events over the preceding year. During the follow-up period, information on medications (if changed from baseline) and the results of examinations performed during routine clinical visits (BP monitoring [office BP, home BP, or ambulatory BP], laboratory testing, echocardiography, vascular function tests, or InBody [InBody S10, Seoul, Korea; a bioelectrical impedance device to measure body composition]) are collected from medical records. Data are collected electronically and transferred to a central electronic data capture system via the internet.

## **Data processing**

After each 7-day home BP measurement period, BP readings stored in the memory of the nocturnal HBPM device are retrieved at the participating institutions. Once the BP data have been imported into the study website, a BP report for each individual, including a summary of the BP data and graphical representation of the BP trend, is automatically generated.

Data management and statistical analyses are conducted at an independent facility (Jichi Medical University Center of Global Home and Ambulatory BP Analysis [GAP], Jichi Medical University Center of Excellence Community Medicine Cardiovascular Research and Development [JCARD], Shimotsuke, Japan).

## **Quality control and reproducibility of echocardiographic measurements**

To ensure consistency of echocardiographic measurements across participating centers, a comprehensive quality control program was implemented. All echocardiographic examinations were performed by certified sonographers (Registered Cardiac Sonographers) or board-certified cardiologists with expertise in echocardiography. Image acquisition and quantitative measurements were conducted independently. All echocardiographic measurements were analyzed by board-certified cardiologists and/or experienced echocardiography specialists who were blinded to clinical information and were not involved in image acquisition. Interobserver, intraobserver, and interinstitution reproducibility were evaluated using a randomly selected subset of echocardiographic datasets obtained from this multicenter study. For interobserver variability, two independent readers from the same institution analyzed the same echocardiographic datasets. Intraobserver variability was assessed by repeat measurements performed by the same reader at separate time points. Interinstitution reproducibility was evaluated by reanalysis of studies that had originally been measured at participating institutions and subsequently remeasured at a central laboratory. Measurement reproducibility was assessed using intraclass correlation coefficients (ICCs), calculated with a two-way random-effects model with absolute agreement (ICC[2,1]) and coefficients of variation (CVs). Acceptable reproducibility was predefined as an ICC >0.85 for left ventricular ejection fraction and volumes and a CV <10% for key echocardiographic parameters, in accordance with previously published quality standards for multicenter echocardiographic studies.

## **Outcome determination**

Annual follow-ups are ongoing to determine the incidence of fatal and nonfatal cardiovascular events.

The following cardiovascular disease events are included:

- **Stroke (including cerebral infarction, cerebral hemorrhage, and subarachnoid hemorrhage)**

The criteria of stroke include sudden onset of neurological deficit persisting for at least 24 h in the absence of any other disease that could account for the symptoms based on the findings of brain computed tomography, and/or magnetic resonance imaging or autopsy (transient ischemic attacks, in which the neurological deficit was completely resolved within 24-h of the onset of symptoms, were not counted as stroke events)

- **Coronary artery disease (acute myocardial infarction, angina pectoris requiring percutaneous coronary intervention, and sudden death within 24 h of the abrupt onset of symptoms)**

The criteria for myocardial infarction include definite electrocardiographic findings [i.e., ST elevation], typical or atypical symptoms and electrocardiographic findings and abnormal enzymes such as troponin T/I, or typical symptoms and abnormal cardiac enzymes with or without electrocardiographic findings). Angina pectoris requiring percutaneous coronary intervention is recorded when coronary revascularization (e.g., percutaneous coronary intervention and coronary artery bypass graft) is needed for suspected myocardial infarction or diagnosed angina pectoris.

- **Aortic dissection**

Diagnosis of aortic dissection is confirmed by identifying dissections involving the aorta on whole-body contrast-enhanced CT.

- **Heart failure**

The criteria of heart failure are an event requiring hospital admission due to clinical manifestations of heart failure, such as the presence of dyspnea, systemic edema or edema in the lower limbs, third heart sound, pulmonary congestion or cardiac dilation on chest X-ray, reduced systolic/diastolic function on echocardiography, and requirement for treatment or intensification of heart failure treatment for worsening heart failure as defined by: (1) addition of new heart failure drugs for worsening heart failure symptoms and signs, (2) initiation of treatment requiring intravenous administration, (3) increased doses of diuretics lasting more than 4 weeks, or (4) mechanical assistance or assisted circulation procedures (e.g., ventilation, ultrafiltration, hemodialysis, aortic balloon pumping, ventricular assist devices, etc.). Subtypes of heart failure events are defined by ejection fraction (EF) at the time of the event and categorized as reduced, midrange, and preserved EF.

#

# **SUPPLEMENTAL TABLES**

**Supplementary Table S1. Associations between diastolic blood pressure measures and left ventricular mass index**

| BP indices | | Average±SD,  mmHg | Correlation coefficient with LVMI, (*p-*value) | *p*-value* for Fisher’s z test | |
| --- | --- | --- | --- | --- | --- |
|  |  |  |  | vs. office DBP | vs. evening DBP |
| Office BP measured by a brachial device | |  |  |  |  |
|  | Office DBP | 80.4±11.3 | -0.033 (0.250) | - | 0.263 |
| Home BP measured by a wrist device in the sitting position | |  |  |  |  |
|  | Morning DBP | 77.2±10.1 | 0.045 (0.117) | 0.055 | 0.423 |
|  | Evening DBP | 70.5±9.6 | 0.013 (0.664) | 0.263 | - |
| Nighttime home BP measured by a wrist device in the lying position | | |  |  |  |
|  | Average of 4 nighttime DBP readings (2:00, 3:00, and 4:00 AM and 4 h after bedtime) | 63.5±8.8 | 0.080 (0.005) | 0.005 | 0.095 |
|  | Average of 3 fixed-time nighttime DBP readings (2:00, 3:00, and 4:00 AM) | 63.6±8.8 | 0.089 (0.002) | 0.003 | 0.058 |
|  | DBP at 2:00 AM | 62.7±9.2 | 0.088 (0.002) | 0.003 | 0.062 |
|  | DBP at 3:00 AM | 63.5±9.0 | 0.087 (0.002) | 0.003 | 0.066 |
|  | DBP at 4:00 AM | 64.8±9.4 | 0.090 (0.002) | 0.002 | 0.056 |
|  | DBP at 4 h after bedtime | 63.0±9.2 | 0.050 (0.082) | 0.041 | 0.357 |

* p-value for Fisher’s z test comparing correlations.

Home BP and nighttime home BP levels were averaged over 7 days.

BP indicates blood pressure; DBP, diastolic blood pressure; SD, standard deviation; LVMI, left ventricular mass index.

**Supplementary Table S2. Associations between left ventricular mass index and nighttime systolic blood pressure levels in different analysis models**

**(A) Female (n=562)**

| Nighttime SBP level |  | Unadjusted | | Adjusted for  age and office SBP | | Adjusted for age, office SBP, and morning SBP | |
| --- | --- | --- | --- | --- | --- | --- | --- |
|  | n | LVMI, g/m^2^ | *p* | LVMI, g/m^2^ | *p* | LVMI, g/m^2^ | *p* |
| SBP < 100 mmHg | 117 | 71.8±1.7 | - | 72.7±1.8 | - | 74.3±1.8 | - |
| 100 < SBP < 110 mmHg | 193 | 81.5±1.4 | <0.001 | 81.6±1.3 | <0.001 | 82.2±1.3 | 0.004 |
| 110 < SBP < 120 mmHg | 158 | 82.7±1.5 | <0.001 | 82.2±1.5 | <0.001 | 81.5±1.5 | 0.034 |
| 120 < SBP < 130 mmHg | 68 | 82.8±2.3 | 0.001 | 82.5±2.3 | 0.009 | 81.0±2.3 | 0.316 |
| SBP > 130 mmHg | 26 | 87.9±3.7 | <0.001 | 87.3±3.7 | 0.006 | 83.5±3.9 | 0.453 |

**(B) Male (n=656)**

| Nighttime SBP level |  | Unadjusted | | Adjusted for  age and office SBP | | Adjusted for age, office SBP, and morning SBP | |
| --- | --- | --- | --- | --- | --- | --- | --- |
|  | n | LVMI, g/m^2^ | *p* | LVMI, g/m^2^ | *p* | LVMI, g/m^2^ | *p* |
| SBP < 100 mmHg | 142 | 84.3±1.9 | - | 84.0±2.0 | - | 86.2±2.1 | - |
| 100 < SBP < 110 mmHg | 187 | 87.6±1.7 | 1.000 | 87.4±1.7 | 1.000 | 88.3±1.7 | 1.000 |
| 110 < SBP < 120 mmHg | 170 | 87.9±1.8 | 1.000 | 88.1±1.8 | 1.000 | 87.9±1.8 | 1.000 |
| 120 < SBP < 130 mmHg | 88 | 98.4±2.4 | <0.001 | 98.4±2.5 | <0.001 | 97.3±2.6 | 0.023 |
| SBP > 130 mmHg | 69 | 103.3±2.7 | <0.001 | 103.7±2.9 | <0.001 | 100.3±3.2 | 0.009 |

Values are mean±SE. SBP indicates systolic blood pressure; SE, standard error; LVMI, left ventricular mass index.

**Supplementary Table S3. Prevalence of LVH using a uniform LVMI cut-off value of 100 g/m²**

| Nighttime SBP level | Female | | |  | Male | | |
| --- | --- | --- | --- | --- | --- | --- | --- |
|  | n | LVH, n (%) | *p* |  | n | LVH, n (%) | *p* |
| <100 mmHg | 117 | 5 (4.3) | - |  | 142 | 30 (21.1) | - |
| 100< and <110 mmHg | 193 | 30 (15.5) | 0.026 |  | 187 | 49 (26.2) | 1.000 |
| 110< and <120 mmHg | 158 | 28 (17.7) | 0.006 |  | 170 | 44 (25.9) | 1.000 |
| 120< and <130 mmHg | 68 | 13 (19.1) | 0.016 |  | 88 | 36 (40.9) | 0.016 |
| 130 mmHg< | 26 | 5 (19.2) | 0.181 |  | 69 | 28 (40.6) | 0.049 |

SBP indicates systolic blood pressure; LVMI, left ventricular mass index; LVH, left ventricular hypertrophy.

**Supplementary Table S4. Associations between morning systolic blood pressure levels and left ventricular mass index or left ventricular hypertrophy, by sex**

**(A) Female (n=562)**

| Morning SBP level |  | LVMI, g/m^2^ | | Prevalence of LVH | |
| --- | --- | --- | --- | --- | --- |
|  | n | Estimate±SE | *p* | n (%) | *p* |
| SBP < 115 mmHg | 70 | 72.1±2.3 | - | 6 (8.6) | - |
| 115 < SBP < 125 mmHg | 150 | 78.8±1.5 | 0.141 | 24 (16.0) | 1.000 |
| 125 < SBP < 135 mmHg | 172 | 80.1±1.4 | 0.029 | 36 (20.9) | 0.241 |
| 135 < SBP < 145 mmHg | 114 | 83.8±1.8 | <0.001 | 32 (28.1) | 0.013 |
| SBP > 145 mmHg | 56 | 87.6±2.5 | <0.001 | 18 (32.1) | 0.012 |

**(B) Male (n=656)**

| Morning SBP level |  | LVMI, g/m^2^ | | Prevalence of LVH | |
| --- | --- | --- | --- | --- | --- |
|  | n | Estimate±SE | *p* | n (%) | *p* |
| SBP < 115 mmHg | 80 | 84.0±2.6 | - | 5 (6.3) | - |
| 115 < SBP < 125 mmHg | 156 | 85.1±1.8 | 1.000 | 10 (6.4) | 1.000 |
| 125 < SBP < 135 mmHg | 195 | 89.7±1.6 | 0.662 | 18 (9.2) | 1.000 |
| 135 < SBP < 145 mmHg | 129 | 92.0±2.0 | 0.156 | 17 (13.2) | 1.000 |
| SBP > 145 mmHg | 96 | 101.4±2.3 | <0.001 | 24 (25.0) | 0.009 |

Values are mean±SE. SBP indicates systolic blood pressure; SE, standard error; LVMI, left ventricular mass index; LVH, left ventricular hypertrophy.

**Supplementary Table S5.** **Associations between left ventricular mass index or left ventricular hypertrophy and nighttime systolic blood pressure levels in patients with morning home <135 mmHg (n=823)**

**(A) Female (n=392)**

| Nighttime SBP level |  | LVMI, g/m^2^ | | Prevalence of LVH | |
| --- | --- | --- | --- | --- | --- |
|  | n | Estimate±SE | *p* | n (%) | *p* |
| SBP < 100 mmHg | 106 | 70.9±1.7 | - | 7 (6.6) | - |
| 100 < SBP < 110 mmHg | 158 | 81.5±1.4 | <0.001 | 30 (19.0) | 0.020 |
| 110 < SBP < 120 mmHg | 101 | 81.2±1.7 | <0.001 | 25 (24.8) | <0.001 |
| SBP > 120 mmHg | 27 | 76.7±3.3 | 0.710 | 4 (14.8) | <0.001 |

**(B) Male (n=431)**

| Nighttime SBP level |  | LVMI, g/m^2^ | | Prevalence of LVH | |
| --- | --- | --- | --- | --- | --- |
|  | n | Estimate±SE | *p* | n (%) | *p* |
| SBP < 100 mmHg | 127 | 84.5±1.9 | - | 7 (5.5) | - |
| 100 < SBP < 110 mmHg | 151 | 87.1±1.8 | 1.000 | 10 (6.6) | 1.000 |
| 110 < SBP < 120 mmHg | 106 | 87.0±2.1 | 1.000 | 10 (9.4) | 1.000 |
| SBP > 120 mmHg | 47 | 92.9±3.2 | 0.150 | 6 (12.8) | <0.001 |

Values are mean±SE. SBP indicates systolic blood pressure; SE, standard error; LVMI, left ventricular mass index.

**Supplementary Table S6. Blood pressure control status and left ventricular structure by the number of antihypertensive medications**

| Number of antihypertensives | 0  (n=102) | 1  (n=384) | 2  (n=392) | >3  (n=340) | *P for trend* |
| --- | --- | --- | --- | --- | --- |
| *BP uncontrol rate, n (%)* |  |  |  |  |  |
| Office BP > 140/90 | 56 (54.9) | 185 (48.2) | 153 (39.0) | 137 (40.3) | 0.002 |
| Morning BP > 135/85 | 40 (39.2) | 134 (34.9) | 151 (38.5) | 146 (42.9) | 0.082 |
| Evening BP > 135/85 | 23 (22.6) | 60 (15.6) | 56 (14.3) | 58 (17.1) | 0.515 |
| Nighttime BP > 120/70 | 39 (38.2) | 118 (30.7) | 110 (28.1) | 93 (27.4) | 0.048 |
|  |  |  |  |  |  |
| *Female* | n=42 | n=211 | n=182 | n=127 |  |
| LVMI, g/m^2^ | 76.8±17.5 | 78.7±18.0 | 80.2±20.3 | 84.2±20.0 | 0.046 |
| LVH, n (%) | 7 (16.7) | 34 (16.1) | 41 (22.5) | 34 (26.8) | 0.017 |
| *Male* | n=60 | n=173 | n=210 | n=213 |  |
| LVMI, g/m^2^ | 85.2±23.0 | 88.3±21.7 | 87.4±22.1 | 95.5±25.7 | <0.001 |
| LVH, n (%) | 6 (10.0) | 16 (9.3) | 18 (8.6) | 34 (16.0) | 0.056 |

Values are mean±SD, or n (%). BP indicates blood pressure; SD, standard deviation; LVMI, left ventricular mass index; LVH, left ventricular hypertrophy.

**Supplementary Table S7. Reproducibility of left ventricular mass index and ejection fraction measurements**

| Reproducibility | Intraobserver (n=30) | | Interobserver (n=30) | | Interinstitution (n=30) | |
| --- | --- | --- | --- | --- | --- | --- |
| Parameter | ICC[2,1] | CV, % | ICC[2,1] | CV, % | ICC[2,1] | CV, % |
| LVMI, g/m^2^ | 0.982 | 2.40 | 0.989 | 1.90 | 0.913 | 7.72 |
| LVEF, % | 0.914 | 1.99 | 0.893 | 1.89 | 0.870 | 2.42 |

ICC[2,1] indicates intraclass correlation coefficients with a two-way random-effects model with absolute agreement; CV, coefficients of variation; LVMI, left ventricular mass index; LVEF, left ventricular ejection fraction.

# **SUPPLEMENTAL FIGURES**

**Supplementary Figure S1. Study outline of the WISDOM–Night Study and the WISDOM–HMOD Study**


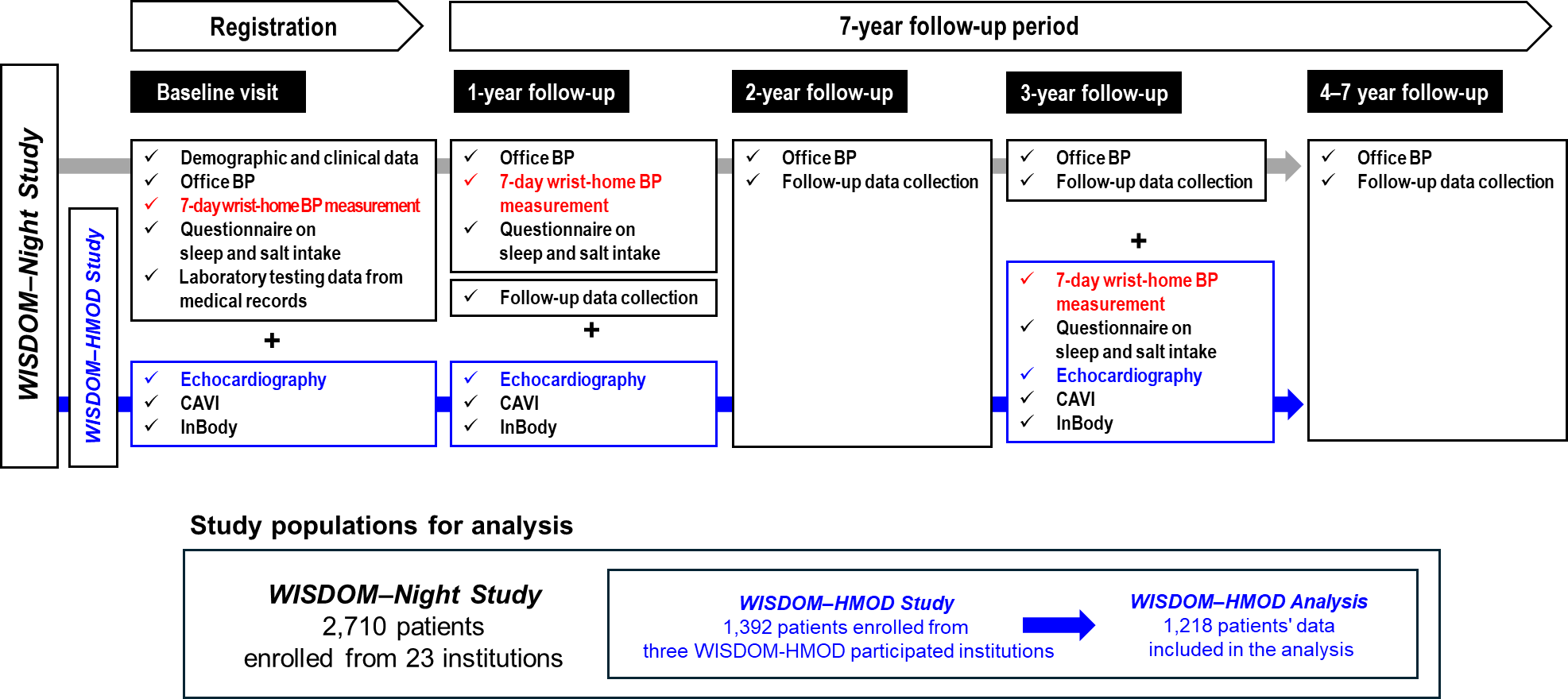


**Supplementary Figure S2. Comparison of nighttime BP average indices**

(A) Comparison of blood pressure values and the prevalence of nocturnal hypertension assessed using the average of four readings (2:00, 3:00, and 4:00 AM and 4 hours after bedtime) versus the average of three fixed-time readings (2:00, 3:00, and 4:00 AM).

(B) Bland–Altman plot of nighttime SBP derived from the average of four readings versus the average of three fixed-time readings.


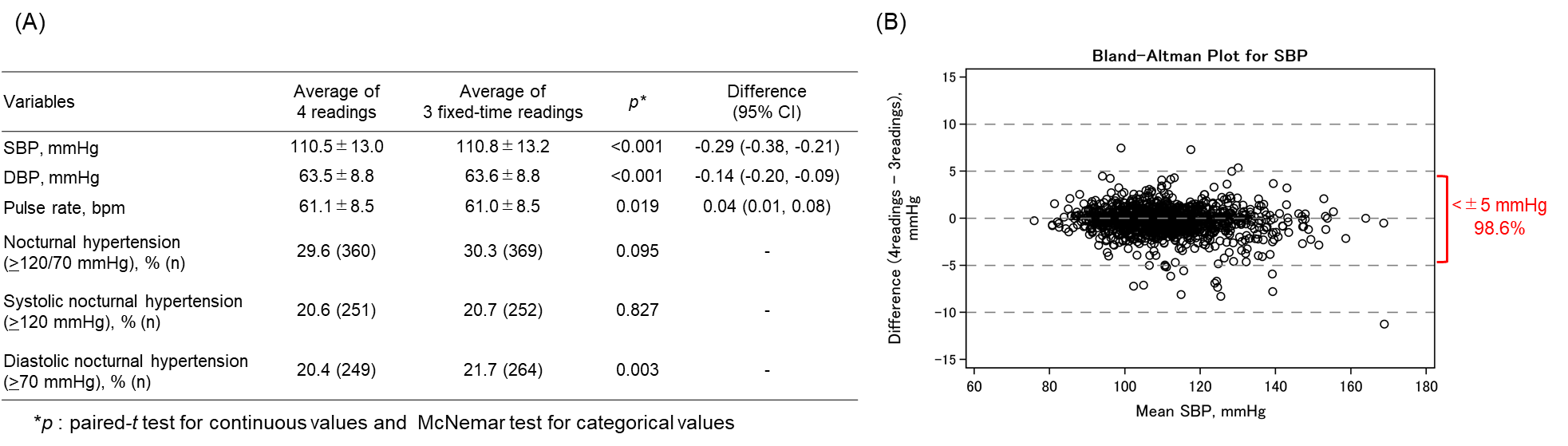

Supplement: Supplementary file 1 [file hyp-83-e26510-s001.docx]
